# Supplementary material for: A scoping review on the use of traditional medicine and oral health in Africa
Source: PLoS One. 2024 May 28;19(5):e0297570. doi: 10.1371/journal.pone.0297570 (PMC11132499; doi:10.1371/journal.pone.0297570)
Supplement: S1 File — (DOCX) [file pone.0297570.s002.docx]

**Supplemental 1: Search term used for the the difference data bases**

| CINAHL = 5 | | |
| --- | --- | --- |
| S/N | Keyword | Search Term |
| 1 | Traditional Medicine | TI ( traditional medicine or complementary and alternative medicine or herb medicine ) OR TI African medicine OR TI african traditional medicine |
| 2 | Oral Health | TI oral health or oral hygiene or dental health or dental care or oral care |
| 3 | Africa | TI africa OR TI african |

| PUBMED = 27 | | |
| --- | --- | --- |
| S/N | Keyword | Search Term |
| 1 | Traditional Medicine | "traditional medicine"[Title/Abstract] OR "traditional medicine"[Title/Abstract] OR "folk remedies"[Title/Abstract] OR "folk remedy"[Title/Abstract] OR "remedies folk"[Title/Abstract] OR "remedy folk"[Title/Abstract] OR "medicine primitive"[Title/Abstract] OR "primitive medicine"[Title/Abstract] OR "medicine folk"[Title/Abstract] OR "folk medicine"[Title/Abstract] OR "medicine indigenous"[Title/Abstract] OR "indigenous medicine"[Title/Abstract] OR "home remedies"[Title/Abstract] OR "home remedy"[Title/Abstract] OR "remedies home"[Title/Abstract] OR (("remediable"[All Fields] OR "remedial"[All Fields] OR "remediate"[All Fields] OR "remediated"[All Fields] OR "remediates"[All Fields] OR "remediating"[All Fields] OR "remediation"[All Fields] OR "remediations"[All Fields] OR "remediative"[All Fields] OR "remediator"[All Fields] OR "remediators"[All Fields] OR "remedied"[All Fields] OR "Remedies"[All Fields] OR "Remedy"[All Fields] OR "remedying"[All Fields]) AND "Home"[Title/Abstract]) OR "Ethnomedicine"[Title/Abstract]  "herbal medicine"[Title/Abstract] OR "traditional medicine"[Title/Abstract] |
| 2 | Oral Health | "oral health"[Title/Abstract] OR "health oral"[Title/Abstract] |
| 3 | Africa | "Africa"[Title/Abstract] |

| Web of Science= 417 | | |
| --- | --- | --- |
| S/N | Keyword | Search Term |
| 1 | Traditional Medicine | ((((((((((((((((TS=(Traditional Medicine)) OR TS=(Herbal medicine)) OR TS=(Folk Remedies)) OR TS=(Folk Remedy)) OR TS=(Remedies, Folk)) OR TS=(Remedy, Folk)) OR TS=(Medicine, Primitive)) OR TS=(Primitive Medicine)) OR TS=(Medicine, Folk)) OR TS=(Folk Medicine)) OR TS=(Medicine, Indigenous)) OR TS=(Indigenous Medicine)) OR TS=(Home Remedies)) OR TS=(Home Remedy)) OR TS=(Remedies, Home)) OR TS=(Remedy, Home)) OR TS=(Ethnomedicine) and Preprint Citation Index (Exclude – Database) |
| 2 | Oral Health | TS=(Oral Health) and Preprint Citation Index (Exclude – Database) |
| 3 | Africa | Preprint Citation Index (Exclude – Database) and NIGERIA or SOUTH AFRICA or ETHIOPIA or KENYA or CAMEROON or UGANDA or TANZANIA or GHANA or EGYPT or ALGERIA or ZIMBABWE or BENIN or SUDAN or MOZAMBIQUE or SENEGAL or TUNISIA or ZAMBIA or COTE IVOIRE or MADAGASCAR or MALI or MAURITIUS or NAMIBIA or NIGER or RWANDA or SCOTLAND or SENEGAMBIA or SIERRA LEONE or CHAD or GAMBIA or LIBERIA or LIBYA or MALAWI or SOUTH SUDAN or SOMALIA or REP CONGO or GUINEA or GABON or TOGO or BURKINA FASO or DEM REP CONGO or MOROCCO (Countries/Regions)  TS=(Africa) and Preprint Citation Index (Exclude – Database) |

| Scopus = 124 | | |
| --- | --- | --- |
| S/N | Keyword | Search Term |
| 1 | Traditional medicine | (TITLE-ABS-KEY ( traditional AND medicine ) OR TITLE-ABS-KEY ( complemetary AND medicine ) OR TITLE-ABS-KEY ( alternative AND medicine ) OR TITLE-ABS-KEY ( herbal AND medicine |
| 2 | Africa | AND TITLE-ABS-KEY ( africa ) |
| 3 | Oral health | AND TITLE-ABS-KEY ( oral AND health ) ) |
