# Supplementary material for: A scoping review on the use of traditional medicine and oral health in Africa
Source: PLoS One. 2024 May 28;19(5):e0297570. doi: 10.1371/journal.pone.0297570 (PMC11132499; doi:10.1371/journal.pone.0297570)
Supplement: Attachment — Submitted filename: PONE-D-23-33702_reviewer.pdf [file pone.0297570.s003.pdf]

PLOS ONE

A scoping review on the use of traditional medicine and oral health in Africa

--Manuscript Draft--

**Manuscript Number:**	PONE-D-23-33702
**Article Type:**	Research Article
**Full Title:**	A scoping review on the use of traditional medicine and oral health in Africa
**Short Title:**	Traditional medicine and oral health
**Corresponding Author:**	Morenike Oluwatoyin Folayan, FWACS<br>Obafemi Awolowo University<br>Ile-Ife, Osun State NIGERIA
**Keywords:**	Ethnobotanical survey; Plant-based remedies; Microorganisms; Dental caries; Periodontal diseases; Halitosis; Oral cancer; Oral healthcare professionals; Financial resources
**Abstract:**	Background<br><br>This review aimed to chart the landscape of literature concerning the precise applications of traditional medicine in managing specific oral diseases and, in doing so, to pinpoint knowledge gaps surrounding the use of traditional medicine for oral disease management in the African context.<br><br>Methods<br><br>A systematic search of the literature was conducted on PubMed, Web of Science, Scopus, and CINAHL. The search was conducted from the inception of the database till September 2023. A search of related citations and references was also carried out. Only English language publications were included. A summary of studies that met the inclusion criteria was conducted.<br><br>Results<br><br>Of the 584 records identified, 11 were duplicates and 12 studies, published between 2006 and 2021, met the inclusion criteria. The studies were published from eight countries located in the five sub-regions on the continent. All the studies were either experimental designs or ethnobotanical surveys and they all utilized plant-based remedies. The five experimental studies aimed to assess the impact of whole plants or plant extracts on the three microorganisms responsible for dental caries and seven responsible for periodontal diseases. The seven ethnobotanical surveys identified 29 to 62 different species from 15 to 29 different families used as topical applications or ingested, for the treatment of tooth and soft tissue related diseases including halitosis and oral cancer.<br><br>Conclusion<br><br>Given the increasing prevalence of oral diseases within the region, the shortage of oral healthcare professionals and limited access to financial resources, it becomes imperative to support the generation of empirical evidence to enhance the provision of traditional medicine for oral healthcare in Africa.
**Order of Authors:**	Moréniké Oluwátóyìn Foláyan, FWACS
	Mobolaji Timothy Olagunju
	Olunike Rebecca Abodunrin
	Omolola Titilayo Alade
**Additional Information:**	
**Question**	**Response**

Powered by Editorial Manager® and ProduXion Manager® from Aries Systems Corporation

**Financial Disclosure**<br><br>Enter a financial disclosure statement that describes the sources of funding for the work included in this submission. Review the submission guidelines for detailed requirements. View published research articles from *PLOS ONE* for specific examples.<br><br>This statement is required for submission and **will appear in the published article** if the submission is accepted. Please make sure it is accurate.<br><br>**Funded studies**<br>Enter a statement with the following details:<br>• Initials of the authors who received each award<br>• Grant numbers awarded to each author<br>• The full name of each funder<br>• URL of each funder website<br>• Did the sponsors or funders play any role in the study design, data collection and analysis, decision to publish, or preparation of the manuscript?<br><br>Did you receive funding for this work?	The author(s) received no specific funding for this work.
**Competing Interests**<br><br>Use the instructions below to enter a competing interest statement for this submission. On behalf of all authors, disclose any competing interests that could be perceived to bias this work—acknowledging all financial support and any other relevant financial or non-financial competing interests.<br><br>This statement is required for submission and **will appear in the published article** if the submission is accepted. Please make sure it is accurate and that any funding sources listed in your Funding Information later in the submission form are also declared in your Financial Disclosure statement.	The authors have declared that no competing interests exist.

View published research articles from *PLOS ONE* for specific examples.<br><br>**NO authors have competing interests**<br><br>Enter: *The authors have declared that no competing interests exist.*<br><br>**Authors with competing interests**<br><br>Enter competing interest details beginning with this statement:<br><br>*I have read the journal's policy and the authors of this manuscript have the following competing interests: [insert competing interests here]*<br><br>* typeset	
**Ethics Statement**<br><br>Enter an ethics statement for this submission. This statement is required if the study involved:<br><br>• Human participants<br>• Human specimens or tissue<br>• Vertebrate animals or cephalopods<br>• Vertebrate embryos or tissues<br>• Field research<br><br>Write "N/A" if the submission does not require an ethics statement.<br><br>General guidance is provided below. Consult the submission guidelines for detailed instructions. **Make sure that all information entered here is included in the Methods section of the manuscript.**	N/A

Powered by Editorial Manager® and ProduXion Manager® from Aries Systems Corporation

**Format for specific study types**<br><br>**Human Subject Research (involving human participants and/or tissue)**<br>• Give the name of the institutional review board or ethics committee that approved the study<br>• Include the approval number and/or a statement indicating approval of this research<br>• Indicate the form of consent obtained (written/oral) or the reason that consent was not obtained (e.g. the data were analyzed anonymously)<br><br>**Animal Research (involving vertebrate animals, embryos or tissues)**<br>• Provide the name of the Institutional Animal Care and Use Committee (IACUC) or other relevant ethics board that reviewed the study protocol, and indicate whether they approved this research or granted a formal waiver of ethical approval<br>• Include an approval number if one was obtained<br>• If the study involved *non-human primates*, add *additional details* about animal welfare and steps taken to ameliorate suffering<br>• If anesthesia, euthanasia, or any kind of animal sacrifice is part of the study, include briefly which substances and/or methods were applied<br><br>**Field Research**<br><br>Include the following details if this study involves the collection of plant, animal, or other materials from a natural setting:<br>• Field permit number<br>• Name of the institution or relevant body that granted permission	
**Data Availability**<br><br>Authors are required to make all data underlying the findings described fully available, without restriction, and from the time of publication. PLOS allows rare exceptions to address legal and ethical concerns. See the PLOS Data Policy and FAQ for detailed information.	Yes - all data are fully available without restriction

A Data Availability Statement describing where the data can be found is required at submission. Your answers to this question constitute the Data Availability Statement and **will be published in the article**, if accepted.<br><br>**Important:** Stating 'data available on request from the author' is not sufficient. If your data are only available upon request, select 'No' for the first question and explain your exceptional situation in the text box.<br><br>Do the authors confirm that all data underlying the findings described in their manuscript are fully available without restriction?	
**Describe where the data may be found in full sentences. If you are copying our sample text, replace any instances of XXX with the appropriate details.**<br><br>• If the data are **held or will be held in a public repository**, include URLs, accession numbers or DOIs. If this information will only be available after acceptance, indicate this by ticking the box below. For example: *All XXX files are available from the XXX database (accession number(s) XXX, XXX.)*.<br>• If the data are all contained **within the manuscript and/or Supporting Information files**, enter the following: *All relevant data are within the manuscript and its Supporting Information files.*<br>• If neither of these applies but you are able to provide **details of access elsewhere**, with or without limitations, please do so. For example:<br><br>*Data cannot be shared publicly because of [XXX]. Data are available from the XXX Institutional Data Access / Ethics Committee (contact via XXX) for researchers who meet the criteria for access to confidential data.*<br><br>*The data underlying the results presented in the study are available from (include the name of the third party*	All relevant data are within the manuscript and its Supporting Information files.

and contact information or URL).<br>• This text is appropriate if the data are owned by a third party and authors do not have permission to share the data.<br><br>* typeset	
Additional data availability information:	

Manuscript

Click here to access/download;Manuscript;Traditional Medicine and oral Health.docx

A scoping review on the use of traditional medicine and oral health in Africa

Morẹ́nikẹ́ Oluwátóyìn Foláyan,[1,2] Mobolaji Timothy Olagunju[3]; Olunike Rebecca Abodunrin[4]; Omolola Titilayo Alade[1,5]

1. Oral Health Initiative, Nigerian Institute of Medical Research, Yaba, Lagos, Nigeria
2. Department of Child Dental Health, Obafemi Awolowo University, Ile-Ife
3. Department of Epidemiology and Biostatistics, Nanjing Medical University, Jiangsu Province, China.
4. Lagos State Health Management Health Agency, Lagos State.
5. Department of Preventive and Community Dentistry, Obafemi Awolowo University, Ile-Ife

Corresponding author
Morenike Oluwatoyin Folayan
Department of Child Dental Health
Obafemi Awolowo University, Ile-Ife
toyinukpong@yahoo.co.uk

Abstract

Background

This review aimed to chart the landscape of literature concerning the precise applications of traditional medicine in managing specific oral diseases and, in doing so, to pinpoint knowledge gaps surrounding the use of traditional medicine for oral disease management in the African context.

Methods

A systematic search of the literature was conducted on PubMed, Web of Science, Scopus, and CINAHL. The search was conducted from the inception of the database till September 2023. A search of related citations and references was also carried out. Only English language publications were included. A summary of studies that met the inclusion criteria was conducted.

Results

Of the 584 records identified, 11 were duplicates and 12 studies, published between 2006 and 2021, met the inclusion criteria. The studies were published from eight countries located in the five sub-regions on the continent. All the studies were either experimental designs or ethnobotanical surveys and they all utilized plant-based remedies. The five experimental studies aimed to assess the impact of whole plants or plant extracts on the three microorganisms responsible for dental caries and seven responsible for periodontal diseases. The seven ethnobotanical surveys identified 29 to 62 different species from 15 to 29 different families used as topical applications or ingested, for the treatment of tooth and soft tissue related diseases including halitosis and oral cancer.

Conclusion

Given the increasing prevalence of oral diseases within the region, the shortage of oral healthcare professionals and limited access to financial resources, it becomes imperative to support the generation of empirical evidence to enhance the provision of traditional medicine for oral healthcare in Africa.

Keywords: Ethnobotanical survey; Plant-based remedies; Microorganisms; Dental caries; Periodontal diseases; Halitosis; Oral cancer; Oral healthcare professionals; Financial resources

Introduction

Traditional medicine encompasses a comprehensive body of knowledge, skills, and practices rooted in the beliefs, experiences, and customs of various cultures [1]. It serves multiple purposes, including the preservation of health, the prevention of ailments, the diagnosis of conditions, and the treatment of physical and mental illnesses [2]. Traditional medicine often employs natural resources, such as plants, animals, fungi, and even geological elements like rocks and minerals, regardless of whether their mechanisms are scientifically understood [3]. This approach is deeply entwined with indigenous beliefs, practical expertise, and ancestral wisdom passed down through generations [4].

Traditional medicine is the primary and sometimes sole recourse for disease prevention and healing in some regions characterized by exclusion and severe poverty and limited access to conventional healthcare services [5]. It plays a vital role in the healthcare landscape of low-income countries, where its utilization is widespread: about 40% to 71% of the population in these regions use traditional medicine [6], and in sub-Saharan Africa, the average prevalence rate of use is 58.2% [7].

While the safe and appropriate use of traditional medicine can offer benefits [8], its concurrent use alongside conventional medical treatments raises concerns regarding its clinical implications [9]. Natural resources and traditional knowledge have played a pivotal role in the development of approximately 40% of pharmaceutical products [10]. As a result, there is a growing interest in exploring the wisdom of ancient cultures, traditional community-based healthcare practices, and natural resources to address contemporary health challenges [ditto].

Traditional medicine is frequently employed in the context of oral healthcare to alleviate toothache, address periodontal inflammation and manage oral mucosal diseases [11,12]. These practices encompass the utilization of medicinal plants for oral hygiene, as well as for their analgesic, antibacterial, antifungal, antiseptic, antimicrobial, antioxidant, and antiviral properties [13]. Nevertheless, there remains a limited understanding of the specific types of traditional medicine used for the management of distinct oral diseases, particularly in Africa where such practices are widespread.

The primary objective of this scoping review is to comprehensively examine the existing body of literature concerning the utilization of traditional medicine for oral healthcare in Africa. This review aims to chart the landscape of literature concerning the precise applications of traditional medicine in managing specific oral diseases and, in doing so, to pinpoint knowledge gaps surrounding the use of traditional medicine for oral disease management in the African context.

Methods

The methodology was based on the Joanna Briggs Institute (JBI) scoping review methodology [14] and reported following the Preferred Reporting Items for Systematic Reviews and Meta-Analyses Extension for Scoping Reviews guidelines (PRISMA-ScR) [15,16].

Identifying research questions

The review was guided by the research questions: what are the published links between traditional medicine practices and oral health in Africa?

Identifying relevant studies

A systematic search of the literature was conducted on PubMed, Web of Science, Scopus, and the Cumulative Index of Nursing and Allied Health Literature (CINAHL) using the terms shown in Appendix 1. The search was conducted from the inception of the database till September 2023. A search of related citations and references was also carried out. No author was contacted.

Study selection

Publications identified through the search strategy were downloaded into Endnote, and imported into Rayyan, and duplicates were removed. Three researchers (ORA, MTO, OTA) performed a screening of the titles and abstracts of the downloaded articles independently using pre-defined inclusion and exclusion criteria. Studies were included if there was an agreement between all the reviewers. Where there were disparities, a fourth reviewer (MOF) was consulted to sort out any disparity between the three reviewers. In addition, three researchers completed the full-text review (ORA, MTO, OTA). Uncertainty regarding whether publications met the inclusion criteria was resolved via consensus among the three researchers or with recourse to the fourth reviewer (MOF).

Inclusion criteria

Peer-reviewed journal articles, books, book chapters, conference proceedings, and reports that cover traditional medicine and oral health focusing on Africa. Studies included were published in the English Language, addressed both preventive and curative aspects of oral health and explored herbal remedies, rituals, or cultural beliefs. Studies reporting outcomes related to the impact of traditional medicine on oral health, including oral diseases, oral hygiene, and oral health-related quality of life were also included. There was no restriction on study design or date of publication.

Exclusion criteria

Animal studies or in vitro experiments were excluded. Also excluded were non-African populations unless they provide crucial comparative insights, unpublished theses and dissertations, letters to the editor, commentaries on studies, scoping, systematic and narrative reviews, and studies whose full lengths cannot be accessed were excluded. Also, studies with insufficient data or methodology not suitable for analysis, and those published on websites were also excluded.

Data charting process

A data-charting form was developed to extract relevant variables. The charted variables were the literature characteristics (e.g., authors, year of publication), study aim, study design, form of traditional medicines used, oral diseases managed, and the outcomes of the studies.

Data analysis

The results of the scoping review were reported according to the PRISMA-ScR checklist. A deductive analysis was conducted using the framework developed for the data extraction. Details generated from the analysis were the years of publication, regions in Africa where studies were conducted, forms of traditional medicines used (plants, plants, animals, fungi, or geological elements), oral diseases (and oral diseases pathogenic organisms) investigated, plants (and plant extracts) investigated, and forms and routes of administration of the traditional medicines for the management of oral diseases.

Results

The search resulted in the identification of 584 records, which were downloaded into Endnote and imported into Rayyan. After de-duplication, 562 records remained. After reviewing titles and abstracts, screening, 26 articles were eligible for full-text screening. On screening the full articles, 13 articles were excluded either because full articles were not available, the focus of the study was not Africa or the study was not related to oral health, leaving 13 articles [17-29] for this review. Figure 1 shows the flow diagram of the publication screening process.

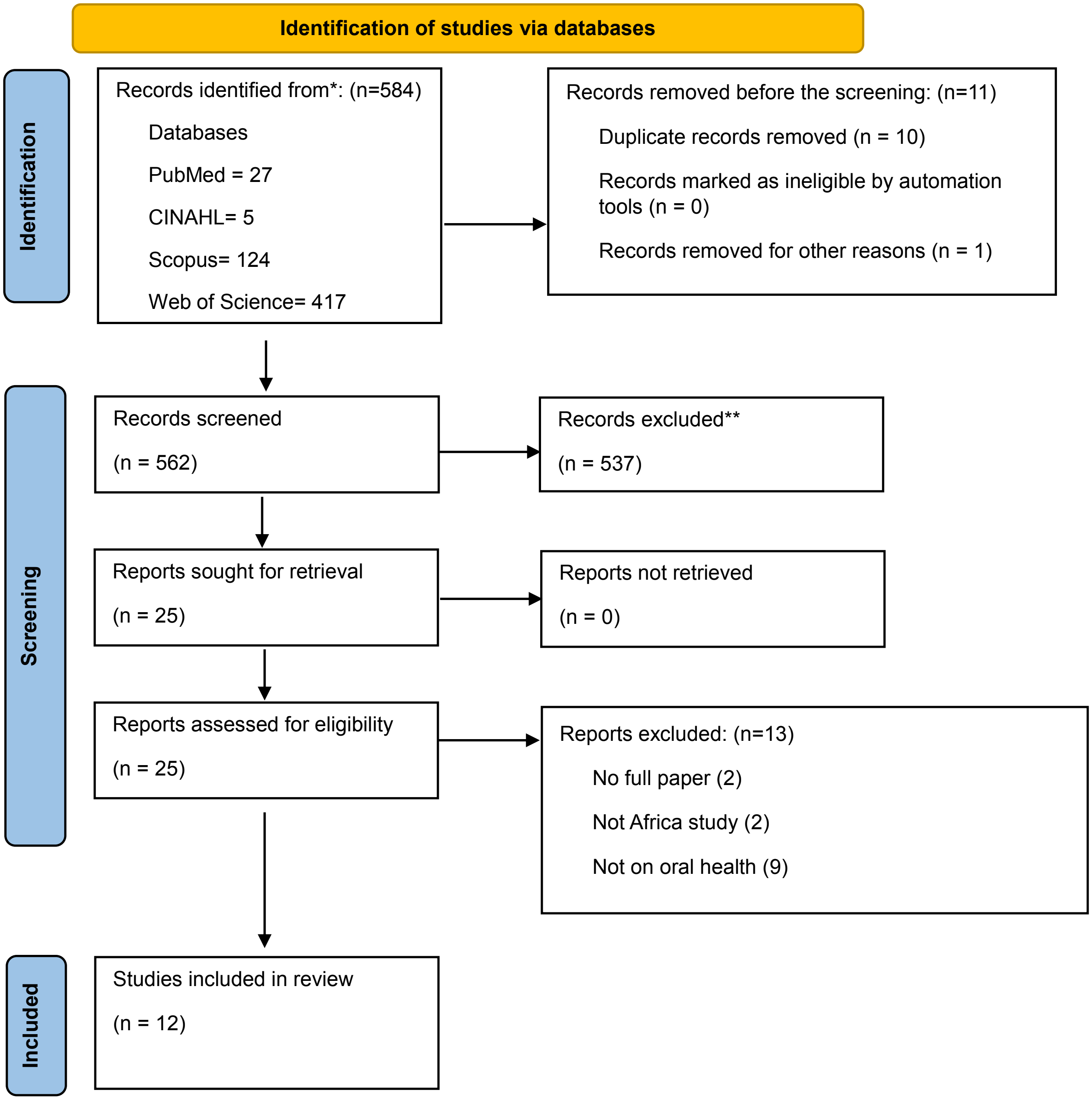

Figure 1: Study Flowchart showing the flow of studies from retrieval to the final included studies

Study characteristics

Table 1 presents an overview of the 12 studies included in this analysis. These studies were conducted over a span of years, ranging from 2006 to 2021. Specifically, three of them were carried out between 2000 and 2010 [25, 27, 28], eight studies were conducted between 2011 and 2020 [17-20, 22-24, 26], and one study was conducted in 2021 [21].

Regarding the geographic distribution of these studies, a quarter of them (25.0%) were conducted in Southern Africa, specifically in South Africa [17, 26, 27]. East Africa was represented by three studies (23.1%): two from Uganda [21, 22] and one from Ethiopia [20]. North Africa accounted for three studies (23.1%): two from Morocco [23, 24] and one from Sudan [19]. West Africa contributed two studies (15.4%): one from Nigeria [25] and one from Burkina Faso [28]. Additionally, one study (8.3%) was conducted in Central Africa, specifically in Cameroon [18].

Study designs

All the studies followed either experimental designs [17, 19, 21, 22, 27] or ethnobotanical surveys [18, 20, 23-26, 28]. All these investigations focused on traditional oral health management practices that primarily utilized plant-based remedies. There was one exception, where minerals were used in combination with plant-based treatments [18].

Characteristics of experimental studies

The experimental studies aimed to assess the impact of whole plants or plant extracts on the microorganisms responsible for dental caries [17, 19, 21, 22, 27] and periodontal diseases [17, 22, 27]. The microorganisms targeted for caries studies included *Streptococcus mutans* [17, 21, 22, 27], S*treptococcus sobrinus* [19, 21], and *Lactobacillus acidophilus* [21, 22]. In periodontopathic organisms studied were *Fusobacterium nucleatum* [17], *Porphyromonas gingivalis* [22, 27], *Aggregatibacter actinomycetemcomitans* [22, 27], *Actinomyces naeslundii* [27], *Actinomyces israelii* [27], *Candida albicans* [27], and *Prevotella intermedia* [27]. Table 2 showed that all the studies reported on the outcome of the activities of the plants on the pathogenic organisms.

Table 1 shed light on the management of various oral diseases using traditional medicines, encompassing a wide spectrum of conditions. These conditions range tooth related diseases: toothache [18, 23-26, 28], dental caries [18, 19, 23, 24, 26], tooth bleaching [18, 23, 24], broken teeth [18], and tooth sensitivity [18, 24]; to soft tissue diseases: mouth sores and ulcers [18, 23-26], abscesses [18, 23, 24, 26], gingivitis and gingival bleeding [18, 23-26, 28], oral infections such as syphilis [18, 23], and mouth thrush [18]. Other oral disorders include halitosis [18, 23, 24, 26], broken jaw [18], temporomandibular joint pain [18], oral cancer [18], tooth extraction [18], and tooth strengthening [26]. There were also traditional medicinal products for oral care [23-25].

Characteristics of ethnobotanical surveys

Table 3 delves into the diverse array of plant-based products used in the management of oral diseases. The number of plant species investigated in these studies varies, ranging from 29 to 62 different species. Furthermore, the studies explore the utilization of plant species from 15 to 29 different families. Plant families prominently used in the management of oral diseases were: Solanaceae [20, 26, 28], Aristolochiaceae [20, 26], Asteraceae [23, 24], Lamiaceae [23, 24], Apiaceae [23, 24], Myrtaceae [24, 28], Rosaceae [23], Anacardiaceae [25], Fabaceae [25], Rutaceae [25], Euphorbiaceae [25], Rhamnaceae [28], Convolvulaceae [28], Caricaceae [28], Verbenaceae [28], Sterculiaceae [28], Scrophulariaceae [28], Rubiaceae [28], Polygalaceae [28], and Poaceae [28].

These traditional medicinal products are derived from various parts of the plants, namely: roots [18, 23-26, 28], leaves [18, 23, 24, 26, 28], stems, twigs, and branches [18, 23-25, 28], barks [23-25, 28], fruits [18, 23, 24], whole plants [18, 24], flowers [23, 24], rhizomes [24, 26], resins [23], umbels [23], seeds [23, 24, 26], saps [23], cloves [24], buds [24], trunks [24], stigmas [24], and bulbs [26].

The modes of administration for these traditional medicinal products encompass a range of practices, namely: topical application of leaves, saps, pastes, or powders [18, 20, 23, 24, 26], mouth rinses [18, 20, 23, 24, 26, 28], gargling [18, 23, 28], brushing [18, 23, 24] and chewing

[18, 23, 25, 26]. There were two systematic routes of administration identified: inhalation [23, 26, 28], and drinking [28].

Table 1: Characteristics of Included Studies

S/no	Title	Author (Year of publication)	Country	Study Aim	Study design
1	Antimicrobial activity and toxicity of extracts from the bark and leaves of South African indigenous Meliaceae against selected pathogens	Oyedeji-Amusa et al., (2020) [17]	South Africa	The study evaluated the antimicrobial efficacy and toxicity of aqueous, methanolic and dichloromethane leaf and bark extracts of South African Meliaceae against selected pathogens. Ocimum basilicum	Experimental
2	Ethnomedicinal plants used by traditional healers to treat oral health problems in Cameroon	Agbor and Naidoo (2015) [18]	Cameroon	To determine the therapeutic methods used by traditional healers to treat oral diseases in Cameroon.	Ethnobotanical survey
3	Anti-caries activity of selected Sudanese Emphasis medicinal plants with on Terminalia laxiflora	Mohieldin et al. (2017) [19]	Sudan	To determine whether T. laxiflora methanolic wood extracts can be used to prevent or treat oral health problems.	Experimental
4	Medicinal plants and their uses by the people in the Region of Randa, Djibouti	Hassan-Abdallah et al. (2013) [20]	Ethiopia	To assess plant-related ethnomedicinal knowledge of the people in Randa Region	Ethnobotanical survey
5	Antibacterial activities of extracts from Ugandan medicinal plants used for oral care	Ocheng et al. (2014) [21]	Uganda	To investigate antibacterial activities of 16 commonly used medicinal plants on microorganisms associated with periodontal diseases and dental caries.	Experimental
6	Essential oils from Ugandan aromatic medicinal plants: chemical composition and growth inhibitory effects on oral pathogens	Ocheng et al., (2015) [22]	Uganda	Assessed the growth inhibitory effects of essential oils extracted from ten Ugandan medicinal plants used traditionally in the management of oral diseases against oral pathogens.	Experimental
7	Oral disorders and ethnobotanical treatments: A field study in the central Middle Atlas (Morocco)	Najem et al. (2020) [23]	Morocco	An inventory of the plants used in the treatment of oral diseases, as well as to document possible risks of intoxication.	Ethnobotanical survey
8	Medicinal and aromatic plants used in traditional treatment of the oral pathology: the ethnobotanical survey in the economic Capital Casablanca, Morocco (north Africa)	Zougagh et al. (2019) [24]	Morocco	To identify the medicinal and aromatic plants most requested for the treatment of the most common oral pathology	Ethnobotanical survey
9	Ethnobotanical plants used for oral healthcare among the Esan tribe of Edo State, Nigeria.	Idu et al. (2009) [25]	Nigeria	To enumerate some plant species and their indigenous uses as chewing sticks for dental and oral healthcare, by the locals of some communities in Esan region of Edo State, Nigeria.	Ethnobotanical survey

10	Medicinal plants used to treat oral diseases in the Lepelle-Nkumpi Municipality, Limpopo Province, South Africa	Semenya (2019) [26]	South Africa	To document the medicinal plants used to treat oral diseases in the Lepelle-Nkumpi Municipality in the Limpopo Province, South Africa.	Ethnobotanical survey
11	Antimicrobial activity of medicinal plants against oral microorganisms	More (2008) [27]	South Africa	To determine the antimicrobial activity of the traditional South African medicinal plants used as chewing sticks against oral microorganisms which are responsible for dental caries	Experimental
12	Use of medicinal plants for the treatment of oral diseases in Burkina Faso	Tapsoba and Deschamps. (2006) [28]	Burkina Faso	To document the use of medicinal plants in the treatment of oral diseases in the Kadiogo province of Burkina Faso.	Ethnobotanical survey

Table 2: Characteristics of the experimental studies included in the scoping review

**S/no**	**Author (Year of publication)**	**Country**	**Form of traditional medicine (no. of products studied)**	**Oral diseases explored**	**Study findings**
1	Oyedeji-Amusa et al., (2020) [17]	South Africa	Plant leaf and bark solvent extracts	Dental caries (*Streptococcus mutans*) Periodontal diseases (*Fusobacterium nucleatum* sub *nucleatum*)	The DCM leaf and bark extracts and methanol leaf extracts of the eight samples showed moderate activity against the 2 pathogens. The methanol bark extract and aqueous extracts showed activities against *S. mutans*.
2	Mohieldin et al. (2017) [19]	Sudan	Plant bark, leaves, wood, and stem	Caries: *S. sobrinus* and glucosyltransferase inhibitory effects	Methanolic extracts of *Terminalia brownii* (bark), *T. laxiflora* (wood), *A. seyal* (bark), Persicaria *glabra* (leaves)and *Tamarix nilotica* (stem) showed good activities against both *S. sobrinus* and glucosyltransferase.
3	Ocheng et al. (2014) [21]	Uganda	Pulp juice and solvent extracts (hexane, methanol and water) from leaves and aerial part.	Dental caries (*Streptococcus mutans, Streptococcus sobrinus, Lactobacillus acidophilus*)	Pulp juice of *Zanthoxylum chalybeum* and *Euclea latidens* showed activity against all the bacteria, Zanthoxylum chalybeum being most active. Hexane extract from aerial part of Helichrysum *odoratissimum* was most active. Methanol extract from leaves of Lantana *trifolia* showed activity against all bacteria
4	Ocheng et al., (2015) [22]	Uganda	Oil extracts	Periodontal disease (*Porphyromonas gingivalis and Aggregatibacter actinomycetemcomitans* Caries (*Streptococcus mutans and Lactobacillus acidophilus*)	Essential oils from the studied plants show marked growth inhibitory effects on periodontopathic A. *actinomycetemcomitans and P. gingivalis,* moderate effects on cariogenic *S. mutans*, and the least effect on *L. acidophilus.*
5	More (2008) [27]	South Africa	Ethanol extracts of eight plants	*Actinobacillus actinomycetemcomitans, Actinomyces naeslundii, Actinomyces israelii, Candida albicans, Porphyromonus gingivalis, Privotella intermedia and Streptococcus mutans*	Six plants exhibited MIC values ranging from 25.0 mg/ml to 0.8 mg/ml. Gram negative bacteria were found to be more resistant to the plant extracts than Gram positive bacteria, except for *Euclea natalensis* which inhibited all three Gram negative bacteria tested in this study

Table 3. Characteristics of the ethnobotanical surveys included in the scoping review

S/no	Author (Year of publication)	Country	Forms in which products are used	Form of traditional medicine	Oral diseases studied	Predominant family of plants used for oral diseases
1	Agbor and Naidoo (2015) [18]	Cameroon	Topical application of paste, mouth rinses, gargling toothbrushing, directing the smoke from melted paste into the mouth, chewing.	Plant roots, leaf, bark, seeds, stems, fruits, whole plants, and minerals as adjunct - sulphur, alum, bicarbonate, calcium carbonate	Toothache, sore mouth, dry mouth, abscess, broken tooth and jaw, tooth sensitivity, mouth thrush, dental caries, gingivitis, oral syphilis, oral cancer, TMJ pain, halitosis, tooth bleaching and dental extraction	52 plants were studied. Information not available
2	Hassan-Abdallah et al. (2013) [20]	Ethiopia	Mouth rinse, topical application of paste	Leaf (as mouth wash or paste)	Dental caries	Aristolochiaceae and Solanaceae were the predominant species used. Informant consensus factors was high for use of some species for mouth diseases (0.93)
3	Najem et al. (2020) [23]	Morocco	Chewing, gargling, topical application of powder or sap, mouth rinse, direct application of leaves, brushing, inhalation,	Resin, flowers, umbels, fruit, seeds, leaves, root, bark, sap, branches	Bad breath, dental caries, gingivitis, dental abscesses, toothache, gingival bleeding, mouth ulcers, herpes labialis, stomatitis, tooth whitening, mouth infection, scurvy, oral care	29 plants from 15 families were studied. The Asteraceae, Lamiaceae, Apiaceae and the Rosaceae were the predominant species used.
4	Zougagh et al. (2019) [24]	Morocco	Direct application, mouth wash, brushing.	Clove, leaves, flowers, seeds, root, bud, bark of root, trunk, stem or fruit, Rhizome, whole plant, stigma	Gingivitis, periodontal abscess, toothache, tooth sensitivity, halitosis, aphtous, ulcers and herpes, teeth cleaning, tooth sparkling, tooth bleaching, caries	46 plants from 22 families. Lamiaceae, Apiaceae, Asteracea and Myrtaceae were the predominant species used. The medicinal ones were dominated by Lamiaceae and Asteracea.
5	Idu et al. (2009) [25]	Nigeria	Chewing	Twigs, root, stem bark, stem, bark, root.	Tooth ache, tooth cleaning, sore gum, gingivitis	32 plants from 23 families were studied. Anacardiaceae, Fabaceae, Rutaceae, and the Euphorbiaceae were the predominant species used.
6	Semenya (2019) [26]	South Africa	Chewing, mouthwash, topically apply extract, inhale, crush leave missed with salt topically applied.	Leaves, roots, seeds, bulb, rhizomes	Bad breath, toothache, dental caries, bleeding gum, dental abscesses, mouth ulcer, swollen gums, strengthen the tooth	41 plants from 30 families<br>The most predominant species reported as remedies for different oral diseases were the Asteraceae and Solanaceae.

7	Tapsoba and Deschamps. (2006) [28]	Burkina Faso	Drinking, mouth-washing, gargling or inhalation	Fresh or dried roots, stems, leaves, and bark	Toothache, gingivitis, bleeding	62 plants from 29 families.<br>The most predominant species reported as remedies for oral diseases were the Rhamnaceae, Convolvulaceae, Myrtaceae, Caricaceae, Verbenaceae, Sterculiaceae, Solanaceae, Scrophulariaceae, Rubiaceae, Polygalaceae and Poaceae.

Discussion

This scoping review provides an overview of traditional medicine practices in oral healthcare across Africa. It encompasses a comprehensive summary of the various types of traditional remedies, their forms, routes of administration, and includes limited insights into experimental studies that assess the properties of plant extracts in combatting pathogenic organisms associated with dental caries and periodontal diseases. The study findings indicates that publications on the use of traditional medicine for the management of oral diseases were from the five sub-regions in Africa, all the studies used plant-based products and the studies were majorly ethnobotanical surveys. All the experimental studies reported on the effects of the plants and plant extracts on the cariogenic or periopathogenic organisms studied. The ethnobotanical studies also indicated the use of plant-based products to manage caries, caries related complications and periodontal diseases in addition to managing other oral diseases such as halitosis, fractured teeth and jaw bones, and the management of oral cancers. The traditional medicine products were majorly used topically through topical applications or mouth rinsing/gargling. There were two reports on systemic applications – ingestion and inhalation.

One of the strengths of this study is its comprehensive overview of research on traditional medicine employed for oral disease management in Africa, a continent known for its significant reliance on traditional healthcare practices for its population's well-being [29]. However, it is important to acknowledge the study's limitations. It primarily focuses on publications available in English and accessible through the specific database utilized for this research. It's worth noting that research publications in Africa encompass a variety of languages, including French and German [29]. Consequently, the inclusion criteria applied in this scoping review may have unintentionally excluded relevant publications on this subject. Despite this limitation, the current study offers valuable insights into the topic at hand.

Firstly, despite the inclusion of 13 publications stemming from research conducted across the five sub-regions of Africa, this number remains disproportionately low for a continent that heavily relies on traditional healthcare practices. These publications are distributed across only eight (17.1%) out of the 56 countries on the continent. It is essential to emphasize the necessity for country-specific publications since traditional medicine is deeply embedded in the unique cultural

contexts of each nation [30]. Moreover, traditional offers a diverse array of health benefits, encompassing both chronic and non-communicable diseases, including those related to oral health [31].

The prevalence of oral diseases is on the rise in Africa and marginalized people are worse affected [32]. In addition, the healthcare resources to address this growing challenge remain scarce across the continent [33]. Consequently, traditional medicines are likely to continue playing a pivotal role in oral disease management in the region for the foreseeable future. To catalyze meaningful improvements in population health, the publication of research findings is imperative. Although there is a noticeable global trend towards increased research publications on traditional medicines, our findings indicate that the rate of growth in publications related to traditional medicine and oral health in Africa does not match this global pace. In addition, the focus has been less on oral health past analysis of publications on traditional medicines from countries in Africa had focused on other diseases like Alzheimer's [34], hypertension, HIV, tuberculosis, malaria, and bleeding disorders [35]. The current study highlights the paramount need to direct attention and provide support to address research on the link between traditional medicines and oral health as a pressing research need area. Doing so can facilitate the development of evidence-based policies that hold the potential to significantly enhance the oral healthcare landscape of countries in the region.

Secondly, the ethnobiological investigations shed light on promising prospects for aligning specific plant families with the management of oral diseases. Our analysis unveiled approximately 20 noteworthy plant families that have been recognized for their potential in oral disease management. However, it is crucial to emphasize the urgency of conducting toxicology studies on this diverse array of plants. This need arises from suggestive evidence indicating that several plants recommended for oral disease treatment may possess inherent dangers and toxicity [23]. To address this concern and pave the way for informed recommendations, further comprehensive studies are imperative. These studies should delve into both the safety and efficacy of traditional medicinal practices employed for oral disease management. By conducting rigorous research, we can ascertain which traditional remedies are both safe and effective for managing specific oral diseases, enabling the formulation of evidence-based guidelines to promote their usage.

Finally, traditional medicine could play a pivotal role in addressing healthcare disparities. Traditional remedies often come at a significantly reduced cost, sometimes up to 50% less expensive than their conventional counterparts. Additionally, the payment arrangements for traditional medicine can be more flexible, with options such as credit or even labor-based compensation. Furthermore, payment for these services may be contingent upon their success [36]. These accessible and adaptable packages offered by traditional medicine providers can make these treatments more appealing, particularly to individuals facing financial hardship. By enhancing the practice of traditional medicine in the realm of oral healthcare, we have the potential to tackle the pressing issues of unequal access to oral health services in low-income African countries [37]. This, in turn, can contribute significantly to achieving the Sustainable Development Goals, particularly Sustainable Development Goal 3, which emphasizes ensuring health and well-being for all, regardless of age or socioeconomic status [38].

Conclusions

This scoping review reveals that there exists a body of literature concerning the utilization of traditional medicine in the management of oral diseases across all the sub-regions of Africa. However, these publications are relatively scarce and originate from less than one-fifth of the countries on the continent. Given the increasing prevalence of oral diseases within the region, coupled with a shortage of oral healthcare professionals and limited access to financial resources, it becomes imperative to provide support for the generation of empirical evidence to enhance the provision of traditional medicine for oral healthcare in Africa. Investments are urgently required to facilitate research on traditional medicine's role in oral healthcare, as well as the translation of research findings into practical applications. These endeavors hold the potential to make a significant contribution towards the achievement of Sustainable Development Goal 3, which aims to ensure good health and well-being for all.

Abbreviations

CINAHL	The Cumulative Index of Nursing and Allied Health Literature
JBI	Joanna Briggs Institute

PRISMA-ScR Preferred Reporting Items for Systematic Reviews and Meta-Analyses Extension for Scoping Reviews guidelines

Declarations

Ethics approval and consent to participate: Not applicable.

Consent for publication: Not applicable.

Availability of data and materials: All data generated or analysed during this study are included in this published article.

Competing interests: Morẹ́nikẹ́ Oluwátóyìn Foláyan is a Senior Editor Board members with BMC Oral Health. All other authors declare no conflict of interest.

Funding: Not applicable.

Authors contributions: MOF conceived the study. The Project was managed by MOF. Data curating was done by ORA, MTO and OTA. Data analysis was conducted by MOF and OTA. MOF developed the first draft of the document. ORA, MTO and OTA read the draft manuscript and made inputs prior to the final draft. All authors approved the final manuscript for submission.

References

1. World Health Organization. Traditional medicine in the WHO South-East Asia Region: review of progress 2014–2019. 2020
2. Chao, J.; Dai, Y.; Verpoorte, R.; Lam, W.; Cheng, Y.-C.; Pao, L.-H.; Zhang, W.; Chen, S. Major Achievements of Evidence-Based Traditional Chinese Medicine in Treating Major Diseases. Biochemical Pharmacology 2017, 139, 94–104. https://doi.org/10.1016/j.bcp.2017.06.123.
3. Miranda, J. J. M. Medicinal plants and their traditional uses in different locations. In Phytomedicine. Academic Press. 2012: 207-223
4. Antonelli, A.; Smith, R. J.; Fry, C.; Simmonds, M. S.; Kersey, P. J.; Pritchard, H. W.; Qi. State of the World's Plants and Fungi (Doctoral Dissertation). Royal Botanic Gardens (Kew) 2020.
5. Mukherjee, P. K. Quality Control and Evaluation of Herbal Drugs: Evaluating Natural Products and Traditional Medicine; Elsevier, 2019.

6. Bodeker, G.; Kronenberg, F. A Public Health Agenda for Traditional, Complementary, and Alternative Medicine. American Journal of Public Health 2002, 92 (10), 1582–1591. https://doi.org/10.2105/ajph.92.10.1582.
7. James, P. B.; Wardle, J.; Steel, A.; Adams, J. Traditional, Complementary and Alternative Medicine Use in Sub-Saharan Africa: A Systematic Review. BMJ Global Health 2018, 3 (5), e000895. https://doi.org/10.1136/bmjgh-2018-000895.
8. Abdel-Aziz, S. M., Aeron, A., & Kahil, T. A. (2016). Health Benefits and Possible Risks of Herbal Medicine. Microbes in Food and Health. 97–116
9. Mohamad Hesam Shahrajabian; Sun, W. Five Important Seeds in Traditional Medicine, and Pharmacological Benefits. Seeds 2023, 2 (3), 290–308. https://doi.org/10.3390/seeds2030022.
10. World Health Organisation. Traditional medicine has a long history of contributing to conventional medicine and continues to hold promise. 10 August 2023. Available at: https://www.who.int/news-room/feature-stories/detail/traditional-medicine-has-a-long-history-of-contributing-to-conventional-medicine-and-continues-to-hold-promise. (accessed 2023-09-29).
11. Kumar, G.; Jalaluddin, M.; Rout, P.; Mohanty, R.; Dileep, C. L. Emerging Trends of Herbal Care in Dentistry. J. Clin. Diagn. Res. 2013, 7 (8), 1827–1829. https://doi.org/10.7860/JCDR/2013/6339.3282.
12. Cohen, L. A.; Bonito, A. J.; Akin, D. R.; Manski, R. J.; Macek, M. D.; Edwards, R. R.; Cornelius, L. J. Toothache Pain: Behavioral Impact and Self-Care Strategies. Special Care in Dentistry 2009, 29 (2), 85–95. https://doi.org/10.1111/j.1754-4505.2008.00068.x.
13. Durgesh Nandini Chauhan; Prabhu Raj Singh; Nagendra Singh Chauhan; Shah, K. Pharmacological Studies in Natural Oral Care; John Wiley & Sons, 2023.
14. Peters, M. D. J.; Marnie, C.; Tricco, A. C.; Pollock, D.; Munn, Z.; Alexander, L.; McInerney, P.; Godfrey, C. M.; Khalil, H. Updated Methodological Guidance for the Conduct of Scoping Reviews. JBI Evidence Synthesis 2020, 18 (10), 2119–2126. https://doi.org/10.11124/jbies-20-00167.
15. Tricco, A. C.; Lillie, E.; Zarin, W.; O’Brien, K. K.; Colquhoun, H.; Levac, D.; Moher, D.; Peters, M. D. J.; Horsley, T.; Weeks, L.; Hempel, S.; Akl, E. A.; Chang, C.; McGowan, J.; Stewart, L.; Hartling, L.; Aldcroft, A.; Wilson, M. G.; Garritty, C.; Lewin, S. PRISMA

Extension for Scoping Reviews (PRISMA-ScR): Checklist and Explanation. Annals of Internal Medicine 2018, 169 (7), 467–473. https://doi.org/10.7326/m18-0850.

16. Prismastatement.org. https://www.prismastatement.org/Extensions/ScopingReviews?AspxAutoDetectCookieSupport=1. (accessed: 30 September 2023).
17. Oyedeji-Amusa, M. O.; Van Vuuren, S.; Van Wyk, B. -.E. Antimicrobial Activity and Toxicity of Extracts from the Bark and Leaves of South African Indigenous Meliaceae against Selected Pathogens. South African Journal of Botany 2020, 133, 83–90. https://doi.org/10.1016/j.sajb.2020.06.032.
18. Ashu Agbor, M.; Naidoo, S. Ethnomedicinal Plants Used by Traditional Healers to Treat Oral Health Problems in Cameroon. Evidence-Based Complementary and Alternative Medicine 2015, 2015, 1–10. https://doi.org/10.1155/2015/649832.
19. Mohieldin, E. A. M.; Muddathir, A. M.; Yamauchi, K.; Mitsunaga, T. Anti-Caries Activity of Selected Sudanese Medicinal Plants with Emphasis on Terminalia Laxiflora. Revista Brasileira de Farmacognosia 2017, 27 (5), 611–618. https://doi.org/10.1016/j.bjp.2017.04.002.
20. Hassan-Abdallah, A.; Merito, A.; Hassan, S.; Aboubaker, D.; Djama, M.; Asfaw, Z.; Kelbessa, E. Medicinal Plants and Their Uses by the People in the Region of Randa, Djibouti. Journal of Ethnopharmacology 2013, 148 (2), 701–713. https://doi.org/10.1016/j.jep.2013.05.033.
21. Ocheng, F.; Bwanga, F.; Joloba, M.; Ann-Karin Borg-Karlson; Gustafsson, A.; Celestino Obua. Antibacterial Activities of Extracts from Ugandan Medicinal Plants Used for Oral Care. Journal of Ethnopharmacology 2014, 155 (1), 852–855. https://doi.org/10.1016/j.jep.2014.06.027.
22. Ocheng, F.; Bwanga, F.; Joloba, M.; Softrata, A.; Azeem, M.; Pütsep, K.; Borg-Karlson, A.-K.; Obua, C.; Gustafsson, A. Essential Oils from Ugandan Aromatic Medicinal Plants: Chemical Composition and Growth Inhibitory Effects on Oral Pathogens. Evidence-Based Complementary and Alternative Medicine 2015, 2015, 1–10. https://doi.org/10.1155/2015/230832.

23. Najem, M.; Hazim Harouak; Ibijbijen, J.; Nassiri, L. Oral Disorders and Ethnobotanical Treatments: A Field Study in the Central Middle Atlas (Morocco). Heliyon 2020, 6 (8), e04707–e04707. https://doi.org/10.1016/j.heliyon.2020.e04707.
24. Zougagh, S.; Belghiti, A.; Rochd, T.; Zerdani, I.; Mouslim, J. Medicinal and Aromatic Plants Used in Traditional Treatment of the Oral Pathology: The Ethnobotanical Survey in the Economic Capital Casablanca, Morocco (North Africa). Natural Products and Bioprospecting 2018, 9 (1), 35–48. https://doi.org/10.1007/s13659-018-0194-6.
25. MacDonald Idu; Umweni, A. A.; T. Odaro; L. Ojelede. Ethnobotanical Plants Used for Oral Healthcare among the Esan Tribe of Edo State, Nigeria. Ethnobotanical Leaflets 2009, 2009 (4), 15.
26. Semenya, S. S.; Sekgothe Mokgoatšana; Maroyi, A. Medicinal Plants Used to Treat Oral Diseases in the Lepelle-Nkumpi Municipality, Limpopo Province, South Africa. J. Pharm. Nutr. Sci. 2019, 9 (3), 175–183. https://doi.org/10.29169/1927-5951.2019.09.03.6.
27. More, G.; Tshikalange, T. E.; Lall, N.; Botha, F.; Meyer, J. J. M. Antimicrobial Activity of Medicinal Plants against Oral Microorganisms. Journal of Ethnopharmacology 2008, 119 (3), 473–477. https://doi.org/10.1016/j.jep.2008.07.001.
28. Tapsoba, H.; Deschamps, J.-P. Use of Medicinal Plants for the Treatment of Oral Diseases in Burkina Faso. Journal of Ethnopharmacology 2006, 104 (1-2), 68–78. https://doi.org/10.1016/j.jep.2005.08.047.
29. Abdullahi, A. A. Trends and Challenges of Traditional Medicine in Africa. African Journal of Traditional, Complementary and Alternative Medicines 2011, 8 (5S). https://doi.org/10.4314/ajtcam.v8i5s.5.
30. Ezema, I. J.; Onyancha, O. B. Open Access Publishing in Africa: Advancing Research Outputs to Global Visibility. African Journal of Library, Archives and Information Science 2017, 27 (2).
31. Zhu, Q.; Kelley, E. F. The WHO Traditional Medicine Strategy 2014-2023: A Perspective. Science 2014, 346 (6216).
32. World Health Organisation. Africa burdened with largest global increase of oral diseases. WHO | Regional Office for Africa. https://www.afro.who.int/news/africa-burdened-largest-global-increase-oral-diseases#:~:text=The%20African%20region%20has%20experienced. (accessed 2023-09-30).

33. Gallagher, J. E.; Mattos Savage, G. C.; Crummey, S. C.; Sabbah, W.; Varenne, B.; Makino, Y. Oral Health Workforce in Africa: A Scarce Resource. International Journal of Environmental Research and Public Health 2023, 20 (3), 2328. https://doi.org/10.3390/ijerph20032328.
34. Masondo, N. A.; Stafford, G. I.; Aremu, A. O.; Makunga, N. P. Acetylcholinesterase Inhibitors from Southern African Plants: An Overview of Ethnobotanical, Pharmacological Potential and Phytochemical Research Including and beyond Alzheimer's Disease Treatment. South African Journal of Botany 2019, 120, 39–64. https://doi.org/10.1016/j.sajb.2018.09.011.
35. Thomford, N.; Dzobo, K.; Chopera, D.; Wonkam, A.; Skelton, M.; Blackhurst, D.; Chirikure, S.; Dandara, C. Pharmacogenomics Implications of Using Herbal Medicinal Plants on African Populations in Health Transition. Pharmaceuticals 2015, 8 (3), 637–663. https://doi.org/10.3390/ph8030637.
36. Debas, H. T.; Ramanan Laxminarayan; Straus, S. E. Complementary and Alternative Medicine. Nih.gov. https://www.ncbi.nlm.nih.gov/books/NBK11796/.
37. Uguru, N.; Onwujekwe, O.; Ogu, U. U.; Uguru, C. Access to Oral Health Care: A Focus on Dental Caries Treatment Provision in Enugu Nigeria. BMC Oral Health 2020, 20 (1). https://doi.org/10.1186/s12903-020-01135-1.
38. World Health Organisation. To achieve Health for All, embrace tradition, innovation and evidence. www.who.int. https://www.who.int/southeastasia/news/opinion-editorials/detail/to-achieve-health-for-all--embrace-tradition--innovation-and-evidence (accessed 2023-09-30).

Click here to access/download
Supporting Information
Appendix 1- Traditional Medicine and Oral Health.docx

Click here to access/download
Supporting Information
PRISMA-ScR-Fillable-Checklist.docx
